# Association of* FMO3* Variants with Blood Pressure in the Atherosclerosis Risk in Communities Study

**DOI:** 10.1155/2019/2137629

**Published:** 2019-02-18

**Authors:** Tyler S. Bryant, Priya Duggal, Bing Yu, Alanna C. Morrison, Tariq Shafi, Georg Ehret, Nora Franceschini, Eric Boerwinkle, Josef Coresh, Adrienne Tin

**Affiliations:** ^1^Department of Epidemiology, Johns Hopkins Bloomberg School of Public Health, Baltimore, MD, USA; ^2^Human Genetics Center, Department of Epidemiology, Human Genetics, and Environmental Sciences, School of Public Health, The University of Texas Health Science Center at Houston, Houston, TX, USA; ^3^Division of Nephrology, Johns Hopkins University School of Medicine, Baltimore, MD, USA; ^4^Center for Complex Disease Genomics, McKusick-Nathans Institute of Genetic Medicine, Johns Hopkins University School of Medicine, Baltimore, MD, USA; ^5^Cardiology, Department of Specialties of Internal Medicine, Geneva University Hospital, Geneva 1211, Switzerland; ^6^Gillings School of Global Public Health, University of North Carolina, Chapel Hill, NC, USA; ^7^Department of Epidemiology, Johns Hopkins Bloomberg School of Public Health and Welch Center for Prevention, Epidemiology and Clinical Research, Johns Hopkins Medical Institutions, Baltimore, MD, USA

## Abstract

*Flavin containing monooxygenase 3* [*FMO3*] encodes dimethylaniline monooxygenase [N-oxide-forming] 3, which breaks down nitrogen-containing compounds, and has been implicated in blood pressure regulation. Studies have reported conflicting results of the association of a common nonsynonymous variant, E158K (rs2266782), with hypertension. We examined the associations of E158K, along with rare and low frequency exonic variants (minor allele frequency [MAF]<5%) in* FMO3* with hypertension, systolic blood pressure (SBP), and diastolic blood pressure (DBP). We included 7,350 European Americans and 2,814 African Americans in the Atherosclerosis Risk in Communities (ARIC) study with exome sequencing of* FMO3*. The association of* FMO3* variants with SBP and DBP was tested using single variant and gene-based tests followed by the replication or interrogation of significant variants in ancestry-specific cohorts based on Bonferroni corrected thresholds. E158K had significant association with higher SBP in African Americans in ARIC (p=0.03), and two low frequency variants had significant association with higher SBP in African Americans (rs200985584, MAF 0.1%, p=0.0003) and European Americans (rs75904274, MAF 1.7%, p=0.006). These associations were not significant with additional samples: E158K in a meta-analysis of SBP of African ancestry (N=30,841, p=0.43) that included ARIC participants and the two low frequency variants in an independent ancestry-specific exome sequencing study of blood pressure (rs200985584, p=0.94; rs75904274, p=0.81). Our study does not support the association of E158K and low frequency variants in* FMO3* with blood pressure and demonstrates the importance of replication in genetic studies.

## 1. Introduction

High levels of trimethylamine N-oxide (TMAO) have been associated with atherosclerotic lesions in mice and various cardiovascular disease (CVD) outcomes (cerebrovascular accident, myocardial infarction, and CVD related mortality) in humans [1]. Flavin Containing Monooxygenase 3 (*FMO3*) regulates the amount of TMAO in the blood through the N-oxygenation of trimethylamine (TMA) [2], which is produced by gut microbiome from dietary L-carnitine and choline. Loss-of-function variants in* FMO3*, such as N61S and P153L, have been found to cause trimethylaminuria (TMAU), a condition characterized by an accumulation of TMA in the blood, due to the decreased catalytic efficiency of FMO3 [3]. Those who have rare variants in* FMO3 *causing trimethylaminuria also commonly have hypertension [4]. Studies in European ancestry have reported conflicting results on the association between E158K (rs2266782), a common missense variant in of* FMO3*, and hypertension. A study in a population of 1,649 Irish participants found no significant association of E158K (minor allele frequency [MAF] = 36%) with hypertension [5]. Another study in a Russian population with 2,995 unrelated participants found that among cigarette smokers, there was an increased risk of hypertension (odds ratio of 1.38) in E158K homozygous individuals (MAF = 45%) [6].

A cohort with large sample size can provide sufficient statistical power to assess the association between E158K and hypertension. In addition, exome sequencing can provide a comprehensive view of all exonic variants in a gene and provide the opportunity to discover novel variants in* FMO3* that may influence blood pressure. The primary aims of the present study were to characterize the number and type of variants located in* FMO3 *in a large cohort of European and African Americans, evaluate the association of the common variant E158K with hypertension reported previously, and detect associations of low frequency* FMO3* variants with systolic blood pressure (SBP) and diastolic blood pressure (DBP).

## 2. Methods 

### 2.1. Study Population

The study population consists of individuals enrolled in the ARIC study, a multisite prospective cohort study aimed to discover predictors of cardiovascular disease outcomes. Detailed methods and description of the ARIC study design have been described previously [7]. The ARIC study enrolled 15,792 participants of ages 45-64 between 1987 and 1989 (visit 1) from four locations across the United States: Forsyth County, North Carolina; Jackson, Mississippi; Minneapolis, Minnesota; and Washington County, Maryland. Participants completed five follow-up visits: visit 2 (1990-92), visit 3 (1993-95), visit 4 (1996-98), visit 5 (2011-13), and visit 6 (2016-18).

The total number of participants with exome sequencing genotype at* FMO3* was 7,810 participants of European ancestry, referred to as European Americans, and 3,180 participants of African Ancestry, referred to as African Americans (Supplementary Figure 1). The following exclusion criteria were applied successively based on quality control measures generated from genotypes obtained from Affymetrix 6.0 microarray on the same participants: participants were excluded from the analysis if they were close based on identity by state (IBS > 0.8) or outliers in genetic principal components (> 8 standard deviation [SD] in European American, n=399, or > 6 SD in African American, n=253). Participants were further excluded if they were missing any phenotype or covariate (European American n=111; African American n=61). Two European American participants in the Jackson, Mississippi study center, were excluded because all other participants in Jackson were African Americans. The Institutional Review Board of all participating institutions approved the ARIC study: University of North Carolina at Chapel Hill, Johns Hopkins University, University of Minnesota, and University of Mississippi Medical Center. All participants provided written informed consent.

### 2.2. Blood Pressure

Hypertension at visit 1 was the primary outcome for this study and was defined as a SBP greater than 140 mmHg, a DBP greater than 90 mmHg, or antihypertensive medication usage [8]. The secondary outcomes were the quantitative measures of SBP and DBP measures at the same visit. Three sitting blood pressure measurements were taken after five minutes of rest during the visit using a random zero sphygmomanometer with an appropriately sized cuff over the brachial artery. Blood pressure measures were calculated as the average of the second and third measurements [8].

### 2.3. Exome Sequencing of* FMO3* and Annotation

Whole exome sequencing was performed using Illumina HiSeq 2000 (Illumina, San Diego, CA) and mapped to the Genome Reference Consortium Human Build 37 (GRCh37). The quality control for* FMO3* exome sequencing data followed the same criteria as reported in Yu et al. [9] After variant calling, variants with posterior probability <0.95, variant read ratio <0.25 or >0.75, or total coverage <10 fold were excluded. A total of 97 variants in* FMO3* passed quality control. No variants located within a 10kb region before and after the gene (positioned at 1q.24.3) were found. Putative function of each variant was annotated using ANNOVAR [10].

### 2.4. Covariates

The clinical covariates were age, sex, body mass index (BMI), and study center. To control for population substructure, we included adjustment for the first 10 principal components generated with EIGENSTRAT using genotypes from whole exome sequencing [9]. Smoking status was shown to interact with the effect of the common variant E158K on hypertension status in a previous study [6]. Therefore, current smoking status was included as a potential effect measure modifier. Hypertension medications can change a person's blood pressure and mask genetic influences. The use of hypertension medications was determined based on the inspection of medications at the study visit along with self-reported confirmation of medication use within two weeks [8]. We imputed the blood pressure values of participants taking antihypertensive medications by adding 15 mmHg to SBP and 10 mmHg to DBP as has been done previously in genome-wide association studies [9, 11].

### 2.5. Data Analysis in the ARIC Study

European and African American participants were analyzed separately. All of the following analyses were adjusted for the first 10 genetic principal components, age, age-squared, sex, BMI, and study center unless otherwise noted. The missense variant E158K was first evaluated for an association with hypertension using multivariate logistic regression in the two ethnic groups separately. Next, to formally test the potential effect measure modification of current smoking status on the variant-hypertension relationship, current smoking status and an interaction term between current smoking status and E158K were added to these ethnic-specific models. Finally, the same analysis was performed stratified by smoking status within the two ethnic groups. An analysis of E158K with continuous SBP and DBP values was also performed using multiple linear regression controlling for the same covariates.

As a secondary analysis, we performed single variant analysis for low frequency variants (MAF between 0.1% and 5%) with SBP and DBP measures as the outcome. Common variants (MAF > 5%) were excluded because rare and low frequency variants may have larger effects on blood pressure and no hypertension associations have been reported for common variants except for E158K. Variants with a MAF < 0.1% were also excluded due to power limitations.

A rank-based inverse normal transformation of blood pressure values was performed as a sensitivity analysis to test the robustness of our results. This analysis prevents any large blood pressure values from influencing the estimates obtained from the single variant analysis by rank-ordering the values to ensure there are no influential points [12]. Gene-based tests, consisting of burden and Sequence Kernel Association Tests (SKAT) that collapse variants in the gene, were performed to determine if* FMO3* was significantly associated with SBP and DBP [13]. These tests included all variants that had MAF < 5%.

### 2.6. Interrogation and Replication

Variants with significant association in the ARIC study were put forward for replication or further interrogation in larger studies. For the E158K association with SBP in African Americans, we interrogated the results from a previously conducted genome-wide association study of 30,841 participants of African ancestry from the Continental Origins and Genetic Epidemiology Network Blood Pressure (COGENT-BP) consortium with ARIC as a participating study [11]. The sample size of the non-ARIC cohorts in this study was large enough to inform the generalizability of the results in ARIC. For two significant low frequency variants, we attempted ancestry-specific replication using data from the Framingham Heart Study (FHS), Cardiovascular Health Study (CHS), and Exome Sequencing Project (ESP). These data are part of the exome sequencing association study of blood pressure from the Cohorts for Heart and Aging Research in Genomic Epidemiology (CHARGE) consortium [14]. ARIC participants in ESP do not overlap with the ARIC participants in our cohort. The statistical significance threshold for interrogation or replication was set at 0.017 (=0.05/3, the number of significant variants in ARIC).

### 2.7. Sensitivity Analysis

Using the same methods described above, sensitivity analyses of the E158K and single variant association with blood pressure were performed stratifying by hypertension medication use to evaluate the effect of adjusting the blood pressure values of those taking antihypertensives. In addition, we performed an analysis of the E158K and single variant associations stratified by sex. A gene-based test as described above was also performed stratified by sex.

### 2.8. Power and Statistical Significance

For the study of the E158K association and the gene-based tests in the ARIC cohort, statistical significance threshold was 0.05. This study of the common variant E158K (MAF of 42% in European Americans and 46% in African Americans) and hypertension had 80% power to detect an odds ratio of 1.11 in European Americans and 1.23 in African Americans. The analysis of E158K with continuous blood pressure values had 99% power to detect a difference in 1.2 mmHg for SBP and 0.7 mmHg for DBP in European Americans and 2.3 mmHg for SBP and 1.4 mmHg for DBP in African Americans. Bonferroni corrected p-values were used to determine significance in the single variant analysis of low frequency variants. At an alpha of 0.05, this study had 80% power in European Americans to detect a change of 13 mmHg, 4 mmHg, and 2 mmHg in SBP and 8 mmHg, 3 mmHg, and 2 mmHg in DBP for MAF of 0.1%, 1%, and 5% respectively. In African Americans, this study had 80% power to detect a change of 24 mmHg, 8 mmHg, and 4 mmHg in SBP and 14 mmHg, 5 mmHg, and 2 mmHg in DBP for MAF of 0.1%, 1%, and 5% respectively. In European Americans, two out of 46 variants had MAF between 0.1% and 5%, therefore the statistical significance threshold for low frequency single variant test was 0.025 (=0.05/2). In African Americans, 14 out of 68 variants had MAF between 0.1% and 5%; therefore the statistical significant threshold was 0.0035 (=0.05/14). Power calculations were performed using QUANTO [15].

### 2.9. Genetic Model

Because the FMO3 activity has been shown to decrease with increasing number of loss-of-function alleles, we used the additive genetic model for all variants [16]. In addition, given that previous association between E158K and hypertension was found using the recessive genetic model, we also performed the analysis using the recessive genetic model for E158K [6]. All analyses were performed in R (www.r-project.org).

## 3. Results

### 3.1. Study Population

In ARIC European Americans, the mean age was 54 with 46% being male (Table 1). The mean SBP and DBP in this population were 118 mmHg and 72 mmHg, respectively. The proportion with hypertension was 26%, and 25% were taking antihypertensive medications. The mean BMI was 27. Around 23% of these participants were self-reported current smokers.

In ARIC African Americans, the mean age was 53, with 37% being male. The mean SBP and DBP were 127 mmHg and 79 mmHg, respectively. The proportion with hypertension was 54%, and 43% were taking antihypertensive medications. The mean BMI was 30, and 28% were self-reported current smokers.

### 3.2. Classification of* FMO3* Variants

There were a total of 97 variants found in the exome sequence of* FMO3* (Table 2). There were 10 intronic variants, 21 synonymous variants, and one variant in the 3′ untranslated region of the gene. Among the 59 nonsynonymous variants, only two were common (MAF ≥ 5%) in African Americans, and three were common in European Americans. In addition, there were four frameshifts and one stopgain and stoploss mutation each. All had MAF < 5%.

### 3.3. Association between E158K and Blood Pressure

In the ARIC study, E158K had a MAF of 42% in European Americans and 46% in African Americans and was in Hardy-Weinberg equilibrium in both populations (p=0.24 and p=0.15, respectively). The association between E158K and hypertension was not significant in both European and African Americans (p>0.05, Table 3). The relationship between E158K and hypertension was not significantly modified by smoking status in both European Americans (p for interaction = 0.41) and African Americans (p for interaction = 0.07). With blood pressure continuous outcome variables, E158K did not have significant association with SBP and DBP in European Americans (p>0.05) but was significantly associated with SBP in African Americans (*β*=1.23 mmHg, p=0.03, Supplementary Table 1). However, no significant association was observed in the large meta-analysis of SBP in African ancestry of the COGENT-BP consortium including ARIC as a participating cohort (N=30,841, *β*=0.13, p=0.43) [11]. In the sensitivity analysis stratified by hypertension medication status, the associations of E158K with SBP and DBP were consistent with the results from the combined analysis (Supplementary Table 2). E158K did not have a significant association with SBP or DBP when stratified by sex (Supplementary Table 3).

### 3.4. Association between Low Frequency Variants and Blood Pressure

**Figure 1:**
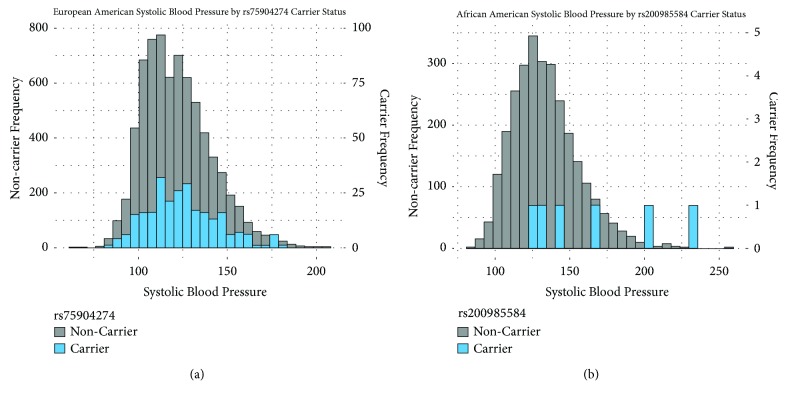
A histogram of minor allele carrier and noncarrier SBP values. Minor allele noncarrier frequency is shown on the left y-axis and minor allele carrier frequency is shown on the right y-axis. (a) shows the SBP values for European Americans who were either minor allele (T) carriers or noncarriers of rs75904274. (b) shows the SBP values for African Americans who were either minor allele (A) carriers or noncarriers of rs200985584.

Exome sequencing found two low frequency variants in European Americans and 14 in African Americans with MAF between 0.1% and 5%. Only one variant was found in both groups (rs75904274). Two nonsynonymous variants were found to have a statistically significant association with SBP separately in African Americans (rs200985584, minor allele count [MAC] = 6, *β*=30.9, p=0.0003, Table 4) and European Americans (rs75904274, MAC=257, *β*=2.98, p=0.006, Table 5).  Figure 1 shows ancestry-specific SBP frequencies in minor allele carriers and noncarriers (with no minor allele) for these two variants. In the sensitivity analysis performed using a rank-based inverse normal transformation of blood pressure values, these two variants remained significant (rs200985584 in African Americans, p=0.0003; rs75904274 in European Americans, p=0.007). No variants were significantly associated with DBP in either European or African Americans. The two variants (N61S and P153L) that were previously reported to be associated with trimethylaminuria were either rare and thus were not included in the association analysis (N61S, rs72549322, MAC=1 in European Americans) or did not have significant association with blood pressure in ARIC (P153L, rs72549326, MAC=38 in European Americans, SBP p=0.83, DBP p=0.68; MAC=2 in African Americans, not included in single variant analysis; all carriers of the minor allele were heterozygotes). In our sensitivity analysis stratified by hypertension medication or sex, there were no additional low frequency variants that had significant association with SBP or DBP (Supplementary Tables 4 and 5).

### 3.5. Aggregate Association of Rare and Low Frequency Variants in* FMO3*

In aggregate,* FMO3* was significantly associated with SBP (SKAT p=0.008) in European Americans including 62 variants with MAF < 5% (Table 6). This result was confirmed by a burden test (*β*=1.74, p=0.04). However, after removing rs75904274 (MAC=257), the significant low frequency variant found in European Americans, the association of* FMO3 *with SBP, was no longer significant in either the SKAT (p=0.74) or the burden test (p=0.76).* FMO3* was not associated with SBP in African Americans in either the SKAT (p=0.34) or the burden test (p=0.43).* FMO3* did not have significant association with DBP in either European or African Americans using the SKAT or burden tests. In our sensitivity analysis stratified by sex, no new significant associations were identified (Supplementary Table 6).

### 3.6. Replication of Significant Associations of the Two Low Frequency Variants with SBP

The replication populations for rs75904274 in European Americans included European ancestry participants of FHS, CHS, and ESP (total N=4,246). A meta-analysis of the association with SBP was not significant (*β*=0.36, p=0.81, Supplementary Table 7). The replication population for rs200985584 in African Americans included participants of African ancestry in ESP (N=1,755) [14]. The association between rs200985584 and SBP also did not replicate in ESP (*β*= -1.1, p=0.94).

## 4. Discussion

In the ARIC study, we found that E158K did not have significant association with hypertension, and the results were similar after stratifying by current smoking status. These results were not driven by differences in MAF because both populations had similar MAFs to previous studies [5, 6]. A significant association of the E158K variant with a 1.23mmHg higher SBP in African Americans was found in the ARIC study, but this association was not significant in a large meta-analysis of SBP in African ancestry including the ARIC study. The associations of rs200985584 with SBP in ARIC African American and rs75904274 with SBP in ARIC European Americans were not replicated in the independent, ancestry-specific populations of FHS, CHS, and ESP.

Two previous studies had conflicting results for the association of E158K with hypertension status [5, 6]. A positive association of E158K with hypertension in current smokers was found in a Russian population [6]. However, the association of E158K and hypertension was not significant in our study in either European Americans or African Americans, even after stratification by current smoking status. Given that our study had 99% power to detect small differences in blood pressure for E158K, the fact that the results were either not significant or unable to be replicated indicates that E158K is not likely to be associated with blood pressure.

Previous studies have found that many individuals presenting with variants in* FMO3 *causing trimethylaminuria also have comorbid hypertension [4–6, 16]. Many low frequency and rare variants have been cited to cause trimethylaminuria in studies of families with the condition [17], prompting the exploration of the hypothesis that rare and low frequency variants (MAF < 5%) in the exome sequence of* FMO3* are associated with hypertension. Our analysis in the ARIC study revealed that most exonic variants in* FMO3* were nonsynonymous, with two of these being associated with higher SBP. However, the replication analysis of these two variants did not show significant associations. Therefore, the significant results in the ARIC study were likely due to chance. These results highlight the ever-important need for replication of significant genetic results.

Measurement errors could affect the associations between variants in* FMO3* and blood pressure. A total of 25% of Europeans and 43% of African Americans included in our study were taking medication for high blood pressure. To account for this, we imputed the blood pressure for these participants by adding 15 mmHg to SBP and 10 mmHg to DBP measures in participants who were on antihypertensive drugs. This imputation may not adequately account for the effects of antihypertension drugs.

## 5. Conclusions

Our study included a large cohort of African and European Americans, providing sufficient power for a definitive study for the association between E158K and hypertension. Contrary to previous report, E158K was not significantly associated with hypertension. In addition, our study examined the association of low frequency and rare variants in* FMO3* with SBP and DBP. The significant associations of rs200985584 and rs75904274 with SBP in the ARIC study were not replicated in ancestry-specific results of other cohorts. Overall, our results do not support the association of E158K and low frequency variants in* FMO3* with blood pressure and demonstrate the importance of replication in genetic studies.

## Figures and Tables

**Table 1:** Baseline characteristics of study populations.

Characteristic	European American	African American
	(n = 7350)	(n = 2814)
Age, years	54.3±5.7	53.2±5.8
Male, %	46.2	37.1
SBP, mmHg	118.2±16.5	127.3±19.5
DBP, mmHg	71.8±9.7	79.4±11.4
Hypertension, %^*∗*^	26.1	54.0
Antihypertensive Use, %	24.8	42.8
BMI, kg/m^2^	27.0±4.8	29.8±6.2
Diabetes, %^†^	8.0	17.7
Current Smoking, %	23.4	28.2

Data presented as mean±standard deviation or percentage.

SBP, systolic blood pressure; DBP, diastolic blood pressure; BMI, body mass index.

^*∗*^Defined as SBP ≥ 140 mmHg, a DBP ≥ 90 mmHg, or antihypertensive medication usage.

^†^Defined as a fasting glucose ≥ 126 mg/dL, non-fasting glucose ≥ 200 mg/dL, or use of antidiabetes mellitus medication.

**Table 2:** Variants found in the *FMO3* exome sequence, stratified by race and minor allele frequency.

*FMO3* Variant Classification	Total Number of Variants	Variants in African Americans		Variants in European Americans	
		MAF <5%	MAF ≥ 5%	MAF < 5%	MAF ≥ 5%
Intronic	10	3	1	6	1
Frameshift	4	1	0	3	0
Nonsynonymous	59	25	2	41	3
Stopgain	1	0	0	1	0
Stoploss	1	1	0	0	0
Synonymous	21	10	2	11	2
UTR3	1	1	0	0	0
*Total*	*97*	*41*	*5*	*62*	*6*

UTR3, 3′ untranslated region; MAF, minor allele frequency.

**Table 3:** Association between E158K in *FMO3* and hypertension, stratified by current smoking status and race in the ARIC study.

	Smokers and non-smokers combined			Smokers only			Non-smokers only		
	N	Odds Ratio	P-value	N	Odds Ratio	P-value	N	Odds Ratio	P-value
		(95% CI)			(95% CI)			(95% CI)	
African Americans	2814	1.14 (0.94, 1.39)	0.18	794	1.42 (0.98, 2.06)	0.07	2020	1.05 (0.84, 1.32)	0.67
European Americans	7350	1.07 (0.93, 1.23)	0.35	1721	1.14 (0.83, 1.58)	0.41	5629	1.05 (0.89, 1.22)	0.58

^*∗*^P-value for interaction between E158K and current smoking status was 0.07 in African Americans and 0.41 in European Americans.

Covariates: age, sex, body mass index, and the first 10 race-specific principal components. The analysis is European Americans also included study center.

**Table 4:** Results from low-frequency single-variant analysis of *FMO3* with SBP or DBP as outcomes in African Americans.

Variant^*∗*^	MAC	Non-coding/coding allele	Classification	SBP			DBP		
				Estimate	P-value	95% CI	Estimate	P-value	95% CI
rs369534680	8	G/T	Intronic	2.89	0.70	(-11.58, 17.35)	4.31	0.34	(-4.45, 13.06)
rs12072582	235	G/C	Nonsynonymous	-0.13	0.92	(-2.84, 2.58)	0.20	0.81	(-1.43, 1.84)
rs75904274	11	G/T	Nonsynonymous	-7.16	0.26	(-19.51, 5.19)	-0.68	0.86	(-8.14, 6.77)
rs144283823	31	G/C	Synonymous	3.53	0.35	(-3.85, 10.90)	1.49	0.51	(-2.96, 5.94)
rs1736557	198	G/A	Nonsynonymous	-1.53	0.31	(-4.51, 1.45)	-0.13	0.88	(-1.93, 1.66)
rs2266780	211	A/G	Nonsynonymous	-0.06	0.97	(-3.01, 2.88	-0.57	0.53	(-2.34, 1.21)
rs115908652	42	C/T	Synonymous	3.01	0.36	(-3.43, 9.45)	1.46	0.46	(-2.42, 5.35)
rs148504519	21	C/T	Synonymous	-6.93	0.13	(-15.87, 2.01)	-1.94	0.48	(-7.33, 3.46)
rs28363581	29	T/C	Nonsynonymous	-3.02	0.42	(-10.39, 4.34)	1.42	0.53	(-3.02, 5.86)
rs2066532	67	G/C	Nonsynonymous	-3.32	0.20	(-8.38, 1.73)	-2.01	0.20	(-5.06, 1.04)
rs79553697	66	T/C	Synonymous	1.64	0.51	(-3.25, 6.53)	1.53	0.31	(-1.42, 4.48)
rs200985584	6	G/A	Nonsynonymous	30.90	0.0003	(14.26, 47.53)	13.22	0.01	(3.16, 23.27)
rs61008738	17	C/T	Nonsynonymous	-9.55	0.06	(-19.48, 0.38)	-2.64	0.39	(-8.63, 3.36)
1:171086503^a^	6	AG/A	Frameshift	1.76	0.84	(-14.99, 18.52)	1.32	0.80	(-8.78, 11.43)

SBP, systolic blood pressure; DBP, diastolic blood pressure; MAC, minor allele count; CI, confidence interval.

^*∗*^All variants in African Americans with a MAF between 0.1% and 5% were included in this analysis.

^a^Chromosome and base pair position in build 37.

**Table 5:** Results from low-frequency single-variant analysis of *FMO3* with SBP and DBP as outcomes in European Americans.

Variant^*∗*^	MAC	Non-coding/coding allele	Classification	SBP			DBP		
				Estimate	P-value	95% CI	Estimate	P-value	95% CI
rs72549326	38	C/T	Nonsynonymous	0.59	0.83	(-4.89, 6.07)	-0.71	0.68	(-4.02, 2.61)
rs75904274	257	G/T	Nonsynonymous	2.98	0.006	(0.85, 5.11)	1.08	0.10	(-0.20, 2.37)

SBP, systolic blood pressure; DBP, diastolic blood pressure; MAC, minor allele count; CI, confidence interval.

^*∗*^All variants in European Americans with a MAF between 0.1% and 5% were included in this analysis.

**Table 6:** Gene-based tests for the association of *FMO3* with SBP and DBP.

Outcome	Test	European Americans		African Americans	
		Estimate	P-value	Estimate	P-value
SBP	SKAT	-	0.008	-	0.34
	Burden	1.74	0.04	-0.57	0.43
DBP	SKAT	-	0.13	-	0.66
	Burden	0.34	0.52	0.17	0.70

SBP, systolic blood pressure; DBP, diastolic blood pressure; SKAT, Sequence Kernel Association Test.

Variants with MAF < 5% were included (European Americans: 62 variants; African Americans: 41 variants).

## Data Availability

The data of the Atherosclerosis Risk in Communities (ARIC) Study used to support the findings of this study have been deposited in the NIH Database of Genotypes and Phenotypes (dbGaP) with Accession no. phs000090.v1.p1. Access is available through dbGaP controlled access application.

## Supplementary Materials

Supplementary Materials Supplementary Figure 1: exclusion flowchart for exome sequencing data from the ARIC study. Supplementary Table 1: association of E158K in FM03 with SBP and DBP in the ARIC study. Supplementary Table 2: sensitivity analysis of the association between E158K in FMO3 and hypertension stratified by medication status. Supplementary Table 3: sensitivity analysis of the association between E158K in FMO3 and hypertension stratified by sex. Supplementary Table 4: single variant association stratified by hypertension medication status. Supplementary Table 5: single variant association stratified by sex. Supplementary Table 6: gene-based testing of FMO3 with systolic and diastolic blood pressure values stratified by sex. Supplementary Table 7: replication of the associations of rs75904274 and rs200985584 with SBP.
