# Supplementary material for: Association of FMO3 Variants with Blood Pressure in the Atherosclerosis Risk in Communities Study
Source: Int J Hypertens. 2019 Feb 18;2019:2137629. doi: 10.1155/2019/2137629 (PMC6397986; doi:10.1155/2019/2137629)
Supplement: Supplementary Materials — Supplementary Figure 1: exclusion flowchart for exome sequencing data from the ARIC study. Supplementary Table 1: association of E158K in FM03 with SBP and DBP in the ARIC study. Supplementary Table 2: sensitivity analysis of the association between E158K in FMO3 and hypertension stratified by medication status. Supplementary Table 3: sensitivity analysis of the association between E158K in FMO3 and hypertension stratified by sex. Supplementary Table 4: single variant association stratified by hypertension medication status. Supplementary Table 5: single variant association stratified by sex. Supplementary Table 6: gene-based testing of FMO3 with systolic and diastolic blood pressure values stratified by sex. Supplementary Table 7: replication of the associations of rs75904274 and rs200985584 with SBP. [file 2137629.f1.docx]

**Supplementary Figures and Tables**

N=2

N=111

N=399

N=253

Drop due to missing phenotype or covariate or invalid value in BP

Drop due to center

7810 European Americans

3180 African Americans

Drop due to IBS, DST and principal components from GWAS

2927 African Americans

7411 European Americans

N=61

7350 European Americans

2816 African Americans

N=0

7350 European Americans

2814 African Americans

**Supplementary Figure 1.** **Exclusion flowchart for exome sequencing data from the ARIC study**

**Supplementary Table 1. Association of E158K in *FM03* with SBP and DBP in the ARIC study**

| **Group** | **ARIC** | | | |
| --- | --- | --- | --- | --- |
|  | **N** | **Estimate** | **p-value** | **95% CI** |
| **SBP** |  |  |  |  |
| African American | 2814 | 1.23 | 0.03 | (0.12, 2.34) |
| European Americans | 7350 | 0.013 | 0.96 | (-0.55, 0.58) |
| **DBP** |  |  |  |  |
| African American | 2814 | 0.56 | 0.10 | (-0.10, 1.23) |
| European Americans | 7350 | 0.05 | 0.76 | (-0.29, 0.39) |

**Supplementary Table 2. Sensitivity analysis of the association between E158K in FMO3 and hypertension stratified by medication status**

| **Ethnicity** | **Hypertension medication status** | **Outcome** | **Estimate** | **SE** | **P-value** |
| --- | --- | --- | --- | --- | --- |
| EA | Yes | SBP | 0.04 | 0.57 | 0.95 |
| EA | Yes | DBP | -0.15 | 0.33 | 0.66 |
| EA | No | SBP | -0.15 | 0.28 | 0.57 |
| EA | No | DBP | 0.02 | 0.17 | 0.89 |
| AA | Yes | SBP | -0.07 | 0.83 | 0.93 |
| AA | Yes | DBP | -0.01 | 0.45 | 0.98 |
| AA | No | SBP | 1.93 | 0.62 | 0.002 |
| AA | No | DBP | 0.95 | 0.38 | 0.013 |

Abbreviations: EA, European American; AA, African American; SE, standard error

**Supplementary Table 3. Sensitivity analysis of the association between E158K in FMO3 and hypertension stratified by sex**

| **Ethnicity** | **Sex** | **Outcome** | **Estimate** | **SE** | **P-value** |
| --- | --- | --- | --- | --- | --- |
| EA | Male | SBP | -0.04 | 0.38 | 0.91 |
| EA | Male | DBP | 0.11 | 0.23 | 0.64 |
| EA | Female | SBP | -0.01 | 0.38 | 0.97 |
| EA | Female | DBP | -0.04 | 0.21 | 0.85 |
| AA | Male | SBP | 1.75 | 0.90 | 0.05 |
| AA | Male | DBP | 1.03 | 0.54 | 0.06 |
| AA | Female | SBP | 0.74 | 0.66 | 0.26 |
| AA | Female | DBP | 0.3 | 0.38 | 0.44 |

Abbreviation. EA, European American; AA, African American; SE, standard error

**Supplementary Table 4: Single variant association stratified by hypertension medication status**

| **Ethnicity** | **Hypertension medication status** | **Variant** | **MAC** | **Non-coding/ coding allele** | **Classification** | **SBP** | | | **DBP** | | |
| --- | --- | --- | --- | --- | --- | --- | --- | --- | --- | --- | --- |
|  |  |  |  |  |  | **Estimate** | **SE** | **P-value** | **Estimate** | **SE** | **P-value** |
| AA | Yes | rs369534680 | 2 | G/T | Intronic | 3.12 | 14.42 | 0.83 | 9.48 | 7.78 | 0.22 |
| AA | Yes | rs12072582 | 100 | G/C | Nonsynonymous | 0.78 | 2.09 | 0.71 | 0.62 | 1.13 | 0.58 |
| AA | Yes | rs75904274 | 5 | G/T | Nonsynonymous | -4.56 | 9.15 | 0.62 | 1.53 | 4.92 | 0.76 |
| AA | Yes | [rs144283823](http://www.ncbi.nlm.nih.gov/SNP/snp_ref.cgi?type=rs&rs=rs144283823) | 14 | G/C | Synonymous | 5.39 | 5.50 | 0.33 | 0.79 | 2.96 | 0.79 |
| AA | Yes | rs1736557 | 76 | G/A | Nonsynonymous | -0.78 | 2.39 | 0.74 | -0.36 | 1.29 | 0.78 |
| AA | Yes | rs2266780 | 85 | A/G | Nonsynonymous | -0.04 | 2.30 | 0.99 | -0.96 | 1.24 | 0.44 |
| AA | Yes | [rs115908652](http://www.ncbi.nlm.nih.gov/SNP/snp_ref.cgi?type=rs&rs=rs115908652) | 19 | C/T | Synonymous | 2.32 | 4.75 | 0.63 | 1.45 | 2.55 | 0.57 |
| AA | Yes | [rs148504519](http://www.ncbi.nlm.nih.gov/SNP/snp_ref.cgi?type=rs&rs=rs148504519) | 10 | C/T | Synonymous | -14.66 | 6.48 | 0.02 | -2.91 | 3.49 | 0.40 |
| AA | Yes | rs28363581 | 10 | T/C | Nonsynonymous | 0.12 | 5.92 | 0.98 | 3.66 | 3.18 | 0.25 |
| AA | Yes | [rs2066532](http://www.ncbi.nlm.nih.gov/SNP/snp_ref.cgi?type=rs&rs=rs2066532) | 23 | G/C | Nonsynonymous | -4.26 | 4.30 | 0.32 | -1.40 | 2.31 | 0.55 |
| AA | Yes | [rs79553697](http://www.ncbi.nlm.nih.gov/SNP/snp_ref.cgi?type=rs&rs=rs79553697) | 39 | T/C | Synonymous | -3.96 | 3.08 | 0.20 | -2.01 | 1.66 | 0.22 |
| AA | Yes | [rs200985584](http://www.ncbi.nlm.nih.gov/SNP/snp_ref.cgi?type=rs&rs=rs200985584) | 4 | G/A | Nonsynonymous | 26.21 | 10.18 | 0.01 | 5.55 | 5.49 | 0.31 |
| AA | Yes | rs61008738 | 6 | C/T | Nonsynonymous | -7.10 | 8.35 | 0.40 | -2.56 | 4.49 | 0.57 |
| AA | Yes | 1:171086503^a^ | 4 | AG/A | Frameshift | -8.67 | 10.25 | 0.40 | -5.29 | 5.50 | 0.34 |
| AA | No | rs369534680 | 6 | G/T | Intronic | 7.33 | 7.12 | 0.30 | 5.03 | 4.41 | 0.25 |
| AA | No | rs12072582 | 135 | G/C | Nonsynonymous | -0.68 | 1.52 | 0.65 | 0.09 | 0.94 | 0.92 |
| AA | No | rs75904274 | 6 | G/T | Nonsynonymous | -9.42 | 7.13 | 0.19 | -2.55 | 4.39 | 0.56 |
| AA | No | [rs144283823](http://www.ncbi.nlm.nih.gov/SNP/snp_ref.cgi?type=rs&rs=rs144283823) | 17 | G/C | Synonymous | 0.83 | 4.25 | 0.85 | 1.23 | 2.62 | 0.64 |
| AA | No | rs1736557 | 122 | G/A | Nonsynonymous | -1.04 | 1.62 | 0.52 | 0.74 | 1.00 | 0.46 |
| AA | No | rs2266780 | 126 | A/G | Nonsynonymous | -0.08 | 1.63 | 0.96 | -0.34 | 1.00 | 0.73 |
| AA | No | [rs115908652](http://www.ncbi.nlm.nih.gov/SNP/snp_ref.cgi?type=rs&rs=rs115908652) | 22 | C/T | Synonymous | 1.69 | 3.74 | 0.65 | -0.15 | 2.30 | 0.95 |
| AA | No | [rs148504519](http://www.ncbi.nlm.nih.gov/SNP/snp_ref.cgi?type=rs&rs=rs148504519) | 11 | C/T | Synonymous | -4.31 | 5.27 | 0.41 | -3.51 | 3.24 | 0.28 |
| AA | No | rs28363581 | 19 | T/C | Nonsynonymous | -3.21 | 4.02 | 0.42 | 1.25 | 2.48 | 0.61 |
| AA | No | [rs2066532](http://www.ncbi.nlm.nih.gov/SNP/snp_ref.cgi?type=rs&rs=rs2066532) | 44 | G/C | Nonsynonymous | 0.06 | 2.67 | 0.98 | -0.31 | 1.64 | 0.85 |
| AA | No | [rs79553697](http://www.ncbi.nlm.nih.gov/SNP/snp_ref.cgi?type=rs&rs=rs79553697) | 26 | T/C | Synonymous | 4.30 | 3.43 | 0.21 | 2.85 | 2.11 | 0.18 |
| AA | No | [rs200985584](http://www.ncbi.nlm.nih.gov/SNP/snp_ref.cgi?type=rs&rs=rs200985584) | 2 | G/A | Nonsynonymous | 25.79 | 12.35 | 0.04 | 18.11 | 7.61 | 0.02 |
| AA | No | rs61008738 | 11 | C/T | Nonsynonymous | -7.72 | 5.28 | 0.14 | -0.54 | 3.25 | 0.87 |
| AA | No | 1:171086503^a^ | 2 | AG/A | Frameshift | 5.67 | 12.37 | 0.65 | 3.53 | 7.62 | 0.64 |
| EA | Yes | [rs72549326](http://exac.broadinstitute.org/variant/1-171076952-C-T) | 9 | C/T | Nonsynonymous | 6.53 | 5.72 | 0.25 | 1.81 | 3.30 | 0.58 |
| EA | Yes | rs75904274 | 62 | G/T | Nonsynonymous | 4.55 | 2.20 | 0.04 | 1.91 | 1.27 | 0.13 |
| EA | No | [rs72549326](http://exac.broadinstitute.org/variant/1-171076952-C-T) | 29 | C/T | Nonsynonymous | -0.61 | 2.67 | 0.82 | -0.92 | 1.63 | 0.57 |
| EA | No | rs75904274 | 195 | G/T | Nonsynonymous | 2.66 | 1.04 | 0.01 | 0.93 | 0.64 | 0.14 |

Abbreviations: EA, European American; AA, African American; SE, standard error

^a^ chromosome and base pair position in build 37

**Supplementary Table 5: Single variant association stratified by sex**

| **Ethnicity** | **Sex** | **Variant** | **MAC** | **Non-coding/coding allele** | **Classification** | **Systolic Blood Pressure** | | | **Diastolic Blood Pressure** | | |
| --- | --- | --- | --- | --- | --- | --- | --- | --- | --- | --- | --- |
|  |  |  |  |  |  | **Estimate** | **SE** | **P-value** | **Estimate** | **SE** | **P-value** |
| AA | Male | rs369534680 | 5 | G/T | Intronic | -11.12 | 9.52 | 0.24 | -3.98 | 5.93 | 0.50 |
| AA | Male | rs12072582 | 101 | G/C | Nonsynonymous | -2.49 | 2.16 | 0.25 | 0.17 | 1.33 | 0.90 |
| AA | Male | rs75904274 | 5 | G/T | Nonsynonymous | -11.58 | 9.53 | 0.22 | -5.78 | 5.89 | 0.33 |
| AA | Male | [rs144283823](http://www.ncbi.nlm.nih.gov/SNP/snp_ref.cgi?type=rs&rs=rs144283823) | 10 | G/C | Synonymous | -1.45 | 6.74 | 0.83 | 2.04 | 4.16 | 0.62 |
| AA | Male | rs1736557 | 79 | G/A | Nonsynonymous | -0.91 | 2.46 | 0.71 | -0.85 | 1.52 | 0.58 |
| AA | Male | rs2266780 | 80 | A/G | Nonsynonymous | -0.74 | 2.48 | 0.77 | 0.65 | 1.53 | 0.67 |
| AA | Male | [rs115908652](http://www.ncbi.nlm.nih.gov/SNP/snp_ref.cgi?type=rs&rs=rs115908652) | 13 | C/T | Synonymous | 8.34 | 5.92 | 0.16 | 3.43 | 3.66 | 0.35 |
| AA | Male | [rs148504519](http://www.ncbi.nlm.nih.gov/SNP/snp_ref.cgi?type=rs&rs=rs148504519) | 11 | C/T | Synonymous | -11.20 | 6.43 | 0.08 | -7.06 | 3.97 | 0.08 |
| AA | Male | rs28363581 | 14 | T/C | Nonsynonymous | 1.35 | 5.36 | 0.80 | 5.45 | 3.30 | 0.10 |
| AA | Male | [rs2066532](http://www.ncbi.nlm.nih.gov/SNP/snp_ref.cgi?type=rs&rs=rs2066532) | 33 | G/C | Nonsynonymous | -5.90 | 3.76 | 0.12 | -3.11 | 2.33 | 0.18 |
| AA | Male | [rs79553697](http://www.ncbi.nlm.nih.gov/SNP/snp_ref.cgi?type=rs&rs=rs79553697) | 25 | T/C | Synonymous | 5.21 | 4.11 | 0.21 | 2.93 | 2.54 | 0.25 |
| AA | Male | [rs200985584](http://www.ncbi.nlm.nih.gov/SNP/snp_ref.cgi?type=rs&rs=rs200985584) | 4 | G/A | Nonsynonymous | 45.13 | 10.54 | 2.03E-05 | 14.66 | 6.56 | 0.03 |
| AA | Male | rs61008738 | 7 | C/T | Nonsynonymous | -8.11 | 8.05 | 0.31 | -2.36 | 4.98 | 0.64 |
| AA | Male | 1:171086503^a^ | 1 | AG/A | Frameshift | 13.09 | 21.21 | 0.54 | 12.21 | 13.12 | 0.35 |
| AA | Female | rs369534680 | 3 | G/T | Intronic | 25.55 | 11.94 | 0.03 | 18.15 | 7.10 | 0.01 |
| AA | Female | rs12072582 | 134 | G/C | Nonsynonymous | 1.74 | 1.81 | 0.34 | 0.38 | 1.08 | 0.72 |
| AA | Female | rs75904274 | 6 | G/T | Nonsynonymous | -4.25 | 8.48 | 0.62 | 2.99 | 5.04 | 0.55 |
| AA | Female | [rs144283823](http://www.ncbi.nlm.nih.gov/SNP/snp_ref.cgi?type=rs&rs=rs144283823) | 21 | G/C | Synonymous | 6.03 | 4.53 | 0.18 | 1.10 | 2.70 | 0.68 |
| AA | Female | rs1736557 | 119 | G/A | Nonsynonymous | -1.89 | 1.94 | 0.33 | 0.33 | 1.15 | 0.77 |
| AA | Female | rs2266780 | 131 | A/G | Nonsynonymous | 0.51 | 1.89 | 0.79 | -1.18 | 1.12 | 0.30 |
| AA | Female | [rs115908652](http://www.ncbi.nlm.nih.gov/SNP/snp_ref.cgi?type=rs&rs=rs115908652) | 28 | C/T | Synonymous | 0.87 | 3.95 | 0.83 | 0.61 | 2.35 | 0.79 |
| AA | Female | [rs148504519](http://www.ncbi.nlm.nih.gov/SNP/snp_ref.cgi?type=rs&rs=rs148504519) | 10 | C/T | Synonymous | -2.64 | 6.55 | 0.69 | 3.30 | 3.89 | 0.40 |
| AA | Female | rs28363581 | 15 | T/C | Nonsynonymous | -6.21 | 5.35 | 0.25 | -2.13 | 3.18 | 0.50 |
| AA | Female | [rs2066532](http://www.ncbi.nlm.nih.gov/SNP/snp_ref.cgi?type=rs&rs=rs2066532) | 34 | G/C | Nonsynonymous | -1.26 | 3.58 | 0.73 | -0.81 | 2.13 | 0.70 |
| AA | Female | [rs79553697](http://www.ncbi.nlm.nih.gov/SNP/snp_ref.cgi?type=rs&rs=rs79553697) | 40 | T/C | Synonymous | -0.91 | 3.14 | 0.77 | 0.40 | 1.87 | 0.83 |
| AA | Female | [rs200985584](http://www.ncbi.nlm.nih.gov/SNP/snp_ref.cgi?type=rs&rs=rs200985584) | 2 | G/A | Nonsynonymous | 1.80 | 14.57 | 0.90 | 10.59 | 8.66 | 0.22 |
| AA | Female | rs61008738 | 10 | C/T | Nonsynonymous | -10.41 | 6.55 | 0.11 | -2.60 | 3.90 | 0.50 |
| AA | Female | 1:171086503^a^ | 5 | AG/A | Frameshift | 0.53 | 9.31 | 0.95 | -0.77 | 5.53 | 0.89 |
| EA | Male | [rs72549326](http://exac.broadinstitute.org/variant/1-171076952-C-T) | 17 | C/T | Nonsynonymous | 0.70 | 4.07 | 0.86 | 0.83 | 2.56 | 0.75 |
| EA | Male | rs75904274 | 112 | G/T | Nonsynonymous | 4.14 | 1.59 | 0.01 | 1.19 | 1.00 | 0.24 |
| EA | Female | [rs72549326](http://exac.broadinstitute.org/variant/1-171076952-C-T) | 21 | C/T | Nonsynonymous | 0.17 | 3.86 | 0.97 | -1.88 | 2.26 | 0.40 |
| EA | Female | rs75904274 | 145 | G/T | Nonsynonymous | 2.10 | 1.49 | 0.16 | 1.00 | 0.87 | 0.25 |

Abbreviations: EA, European American; AA, African American; SE, standard error

^a^ chromosome and base pair position in build 37.

**Supplementary Table 6: Gene-based testing of FMO3 with systolic and diastolic blood pressure values stratified by sex**

| Outcome | Test | European Americans | | | | African Americans | | | |
| --- | --- | --- | --- | --- | --- | --- | --- | --- | --- |
|  |  | Men | | Women | | Men | | Women | |
|  |  | Estimate | P-value | Estimate | P-value | Estimate | P-value | Estimate | P-value |
| SBP | SKAT | - | 0.01 | - | 0.24 | - | 0.07 | - | 0.63 |
|  | Burden | 1.74 | 0.17 | 1.74 | 0.15 | -1.37 | 0.24 | -0.11 | 0.90 |
| DBP | SKAT | - | 0.35 | - | 0.32 | - | 0.22 | - | 0.88 |
|  | Burden | 0.21 | 0.79 | 0.41 | 0.56 | 0.21 | 0.77 | 0.08 | 0.89 |

**Supplementary Table 7. Replication of the associations of rs75904274 and rs200985584 with SBP**

| Study | N | Estimate | SE | P-value |
| --- | --- | --- | --- | --- |
|  |  |  |  |  |
| [rs200985584](http://www.ncbi.nlm.nih.gov/SNP/snp_ref.cgi?type=rs&rs=rs200985584) |  |  |  |  |
| ESP (African ancestry) | 1755 | -1.1 | 14.63 | 0.94 |
| rs75904274  (European American) |  |  |  |  |
| FHS | 1227 | 1.58 | 1.89 | 0.40 |
| CHS | 680 | -5.42 | 5.16 | 0.29 |
| ESP (European ancestry) | 2339 | -0.54 | 2.67 | 0.84 |
| Overall | 4246 | 0.36 | 1.48 | 0.81 |
